# INCIDENCE, CHARACTERISTICS, AND OUTCOMES OF MACULAR NEOVASCULARIZATION IN EXTENSIVE MACULAR ATROPHY WITH PSEUDODRUSEN-LIKE APPEARANCE

**DOI:** 10.1097/IAE.0000000000004506

**Published:** 2025-04-30

**Authors:** Andrea Trinco, Alessio Antropoli, Lorenzo Bianco, Chiara Zaffalon, Matteo Airaldi, Alessandro Lanzani, Mariano Cozzi, Alessandro Invernizzi, Alessandro Arrigo, Andrea Saladino, Francesco Bandello, Francesca Bosello, Stefano Casati, Anna Paola Salvetti, Maurizio Battaglia Parodi, Giovanni Staurenghi, Francesco Romano

**Affiliations:** * Eye Clinic, Department of Biomedical and Clinical Sciences, Ospedale Luigi Sacco, University of Milan, Milan, Italy;; † Department of Ophthalmology, IRCCS San Raffaele Scientific Institute, Milan, Italy;; ‡ University Vita-Salute San Raffaele, Milan, Italy;; § Ophthalmic Unit, Department of Neurosciences, Biomedicine and Movement Sciences, University of Verona, Verona, Italy;; ¶ Department of Medical and Surgical Specialties, Radiological Sciences, and Public Health, University of Brescia, Brescia, Italy;; ** St Paul's Eye Unit, Royal Liverpool and Broadgreen University Hospitals, University of Liverpool, Liverpool, UK;; †† Save Sight Institute, University of Sidney, Sidney, NSW, Australia; and; ‡‡ Harvard Retinal Imaging Lab, Retina Service, Department of Ophthalmology, Massachusetts Eye and Ear, Harvard Medical School, Boston, Massachusetts

**Keywords:** EMAP, extensive macular atrophy with pseudodrusen-like appearance, macular neovascularization, MNV, incidence, risk factors, outcomes, CNV

## Abstract

**Purpose:**

To report the incidence, features, and clinical outcomes of macular neovascularization (MNV) in a large Italian cohort of patients with extensive macular atrophy with pseudodrusen-like appearance.

**Methods:**

Retrospective, longitudinal study including 79 extensive macular atrophy with pseudodrusen-like appearance patients (158 eyes) with ≥6 months of follow-up at three retina clinics. Medical records and imaging were reviewed for demographic and clinical data, including age, best-corrected visual acuity (BCVA), MNV features, and retinal pigment epithelium atrophy size, measured by short-wavelength autofluorescence and refined with near-infrared and optical coherence tomography imaging. Main outcomes included cumulative MNV incidence, MNV risk factors, and BCVA and retinal pigment epithelium atrophy changes in eyes with and without MNV.

**Results:**

Over a mean follow-up of 40.4 months, MNV developed in 14 eyes (10 patients), with a 4-year cumulative incidence of 15.2%. Most MNVs were Type 2 (86%) and subfoveal (64%). Cox regression identified younger age, fellow eye involvement, smaller retinal pigment epithelium atrophy size, and greater central subfield thickness (all *P* < 0.01) as significant risk factors for MNV. While eyes with MNV had lower baseline BCVA (58.4 vs. 71.4 letters, approximately 20/63 vs. 20/40 Snellen; *P* = 0.005), BCVA decline over time was similar between the two groups (−3.9 vs. −4.1 letters/year, *P* = 0.69). However, retinal pigment epithelium atrophy progressed faster in MNV eyes (3.4 vs. 2.8 mm^2^/year, *P* = 0.02).

**Conclusion:**

In this extensive macular atrophy with pseudodrusen-like appearance cohort, MNV had a cumulative incidence of 15.2% at 4 years. Although BCVA outcomes were comparable, MNV was associated with faster atrophy progression, potentially because of a more aggressive disease phenotype or fibro-atrophic changes.

## Abstract 2

Supplemental Digital Content is Available in the Text.

We estimated a cumulative incidence of macular neovascularization in extensive macular atrophy with pseudodrusen-like appearance of 15.2% at 4 years (4.2% excluding left-censored data), most being Type 2 lesions and located subfoveally. While best-corrected visual acuity changes were comparable, macular neovascularization eyes showed faster retinal pigment epithelium atrophy progression, suggesting a more aggressive phenotype or increased fibro-atrophic changes.

Extensive macular atrophy with pseudodrusen-like appearance (EMAP) is a severe bilateral retinal degenerative disorder first described in France by Hamel et al.^1^ Extensive macular atrophy with pseudodrusen-like appearance predominantly affects middle-aged women, although onset can vary, and typically leads to legal blindness within 5 years.^1,2^ While its precise etiology remains unclear, the EMAP Case-Control National Clinical Trial identified links with chronic low-dose organophosphates exposure and nonspecific complement system activation.^3,4^

Extensive macular atrophy with pseudodrusen-like appearance diagnosis is primarily clinical, based on a triad of vertically oriented macular atrophy, diffuse pseudodrusen-like deposits, and peripheral paving stones.^1,5,6^ Advances in retinal imaging have identified additional key features, including the separation between the retinal pigment epithelium (RPE) and Bruch membrane (BrM) preceding atrophy, BrM ruptures, and an oval area of relative photoreceptor-RPE sparing, located temporal to the macula.^5,7–10^

Romano et al developed the first EMAP classification based on longitudinal observations over 3 years.^5,11^ Stage 1 is characterized by RPE-BrM separation with few or no confluent areas of atrophy and preserved visual acuity. Stage 2 involves more extensive confluent areas of atrophy without foveal involvement, while Stage 3 presents with significant visual loss because of foveal involvement—either from expansion of macular atrophy or development of subfoveal non-neovascular fibrosis.

Recently, macular neovascularization (MNV) has been reported as a complication of EMAP, contributing to additional visual loss.^5,10,12^ Information on this rare complication is limited to few reports.^13–15^ Romano et al included MNV as a “+” feature in their classification, indicating its possible occurrence at any stage. However, longitudinal data on MNV characteristics and their outcomes remain scarce. This study aims to report the incidence, clinical characteristics, and long-term outcomes of MNV in a large cohort of EMAP patients from three Italian referral clinics.

## Methods

This retrospective, longitudinal study included data from three Italian referral centers for retinal disorders: Ospedale Luigi Sacco (Milan), Ospedale San Raffaele (Milan), and Ospedale Borgo Roma (Verona). The research adhered to the Declaration of Helsinki. Institutional review board approval was obtained from each center, and all participants provided written informed consent.

We reviewed electronic medical records and imaging studies of EMAP patients followed at these clinics between January 2009 and February 2024. Inclusion criteria were 1) diagnosis of EMAP based on the classic clinical triad,^1^ 2) age <55 years at diagnosis or symptoms onset, 3) at least two clinical examinations with retinal imaging over a follow-up of ≥6 months, and 4) negative genetic test for inherited phenocopies of EMAP using next-generation sequencing (Illumina MiSeq; Illumina, San Diego, CA), including screening for late-onset retinal degeneration (C1QTNF5, OMIM #605670), Sorsby fundus dystrophy (TIMP3, OMIM #136900), and pseudoxanthoma elasticum (ABCC6, OMIM #264800).^2,16^ Exclusion criteria included 1) other ocular or systemic conditions affecting the analysis, 2) refractive errors exceeding |6| diopters, 3) media opacities impairing image quality, and 4) history of intraocular inflammation or ocular surgery other than cataract extraction and intravitreal antivascular endothelial growth factor (anti-VEGF) injections.

Retinal imaging required for eligibility included fundus photographs (Sacco: EIDON, CenterVue from 2016; previously FF 450plus, Carl Zeiss; San Raffaele & Borgo Roma: Optos California, Optos plc. from 2015; previously TRC-50DX, Topcon), 30° × 30° short-wavelength autofluorescence and macular spectral-domain optical coherence tomography (SD-OCT; at least 20° × 20°, centered on the fovea) using the Spectralis HRA + OCT system (Heidelberg Engineering GmbH, Heidelberg, Germany). Given the retrospective design, follow-up and treatment strategies were not standardized across centers. However, common protocols were 1) follow-up every 6 to 12 months for patients without MNV, 2) anti-VEGF treatment for active MNV, and 3) monthly monitoring for active MNV cases, with extended intervals (up to 4 months) once inactivity was achieved.

### Study Protocol and Data Collection

Baseline and follow-up data collected included demographic and clinical information: age, sex, smoking status, lens status, best-corrected visual acuity (BCVA, converted to early treatment for diabetic retinopathy study [ETDRS] letters), presence of MNV, anti-VEGF agent used (bevacizumab, ranibizumab, aflibercept), and lesion activity (percentage of visits with active MNV). The frequency of anti-VEGF injections and lesion activity were reported annually, as previously described.^17^

Macular neovascularization diagnosis required identification of a neovascular network on indocyanine green angiography or, if contraindicated, OCT angiography, along with leakage on fluorescein angiography.^18^ Treatment was initiated with either a single intravitreal anti-VEGF injection or a loading dose of three monthly injections, as determined by the treating physician, followed by a *pro re nata* regimen. Re-treatment criteria included 1) persistent or recurrent intra-/subretinal exudation, 2) significant growth or undefined neovascular membrane margins on SD-OCT, 3) hemorrhages on fundus photography, or 4) leakage on fluorescein angiography.^19^

### Imaging Analysis

Imaging studies from all available visits were independently graded by two masked retina specialists (F.R. and M.B.P.) using Heidelberg Eye Explorer software (HEYEX; version 1.10.4.0, Heidelberg Engineering GmbH, Germany). Any qualitative discrepancies and quantitative measurements differing by >10% were resolved by a third senior grader (G.S.).

30° × 30° short-wavelength autofluorescence images were analyzed to measure the size of RPE atrophy and assess the autofluorescence signal at the atrophic borders (iso- or hyperautofluorescent).^2,20^ Specifically, RPE atrophy areas, which appeared hypoautofluorescent, were measured with the semi-automated Heidelberg RegionFinder tool (version 2.6.4) and manually adjusted using co-registered near-infrared (bright hyperreflectivity) and SD-OCT (outer retinal and RPE loss) scans to exclude other hypoautofluorescent lesions (e.g., macular pigments, hemorrhages, and RPE tears).^2,21,22^

The following SD-OCT biomarkers were assessed: 1) presence of any vitreomacular interface disorder^23^; 2) central subfield thickness (CST),^24^ defined as the distance from the internal limiting membrane to BrM within the central 1 mm circle; 3) outer retinal tubulations^25^; and 4) subfoveal choroidal thickness, measured from the outer RPE line to the sclerochoroidal interface.^26^ Non-neovascular fibrosis (Stage 3b) was identified on fundus photographs as a well-demarcated mound of yellowish-white tissue, without exudative signs on SD-OCT or a neovascular network on OCT angiography.^5,27^

For eyes with MNV, baseline images were reviewed to determine the type and location of neovascularization, based on the consensus on neovascular age-related macular degeneration (AMD) nomenclature (Type 1, 2, 3, or polypoidal choroidal vasculopathy) and Comparison of Age-Related Macular Degeneration Treatments Trials study group criteria.^18,28^ Specifically, MNV location was categorized as subfoveal (involving the foveal center), juxtafoveal (1–200 *µ*m from the foveal center), or extrafoveal (>200 *µ*m) using indocyanine green angiography images, obtained 1 to 10 minutes postinjection, and overlaid on near-infrared and SD-OCT scans.^17^

### Statistical Analysis

All analyses were conducted using Stata/MP 18 (StataCorp; College Station, TX) and R Studio Version 2023.12.0 + 369 (RStudio, PBC; Boston, MA). Significance was considered at α < 0.05 for two-sided tests. Descriptive statistics included mean (SD), median (interquartile range, IQR), or frequencies (%), with variable distributions assessed via Shapiro–Wilk test. Intergrader agreement was evaluated using Cohen kappa for categorical measurements and two-way mixed-effects intraclass correlation coefficients for continuous data.

Macular neovascularization incidence was calculated per 100 person-years, and cumulative incidence was presented using Kaplan–Meier estimates (expressed as %), both for the whole cohort and after excluding left-censored data (MNV at baseline). Mixed-effects Cox regression determined the risk factors for MNV development, reported as hazard ratios (HRs). Time-varying variables included age, smoking status, fellow eye involvement, atrophy borders, RPE atrophy size, any vitreomacular interface disorder, CST, SCT, and outer retinal tubulation. Only variables significant in univariable analysis were considered in multivariable models. Linear mixed-effects models assessed the effects of MNV, time, and their interaction term on BCVA and RPE atrophy size, adjusted for baseline values and age. Random intercepts accounted for data hierarchy (eyes nested within patients). Scaling was applied to age (per 0.1), CST (per 0.01), and square-rooted RPE atrophy size to enhance model stability and account for baseline lesion size. Results are reported as mean estimates with 95% confidence intervals.

## Results

Our study included 158 eyes from 79 EMAP patients, predominantly female (52, 65.8%), with a median age of 57.3 years (IQR: 53.8–60.4) at baseline. Of the 176 eyes initially considered (88 patients), 18 eyes from nine patients were excluded because of insufficient follow-up (8 eyes), glaucoma (4 eyes), high refractive errors (4 eyes), and previous subthreshold laser treatment (2 eyes).

The mean baseline BCVA was 70.7 (SD: 21.0) ETDRS letters (approximately 20/40 Snellen), with a median RPE atrophy size of 8.2 mm^2^ (IQR: 2.2–18.0). Table 1 summarizes the demographic and clinical characteristics. Intergrader agreement was substantial-to-good for all variables (see **Supplemental Digital Content 1**, http://links.lww.com/IAE/C568).

**Table 1.** Demographic and Clinical Characteristics of the Analyzed EMAP Cohort

EMAP Group		
Patients (eyes)	79	158
Age years, mean (SD) and median (IQR)	57.2 (5.0)	57.2 (53.8–60.5)
Sex		
Males n (%)	27	34.2%
Females n (%)	52	65.8%
Lens status		
Phakic eyes n (%)	148	93.7%
Pseudophakic eyes n (%)	10	6.3%
Smoking status		
Never n (%)	51	64.6%
Former/current n (%)	5	6.3%
Unknown n (%)	23	29.1%
Baseline BCVA ETDRS letters, mean (SD) and median (IQR)	70.7 (21.0)	80.0 (65.0–84.0)
Snellen equivalent	20/40	20/25
Baseline RPE atrophy size mm^2^, mean (SD) and median (IQR)	11.3 (9.0)	8.2 (2.2–18.0)
Follow-up months, mean (SD) and median (IQR)	40.4 (34.7)	34.4 (8.3–61.3)

### Characteristics of the Macular Neovascularization

**Fig. 1.**
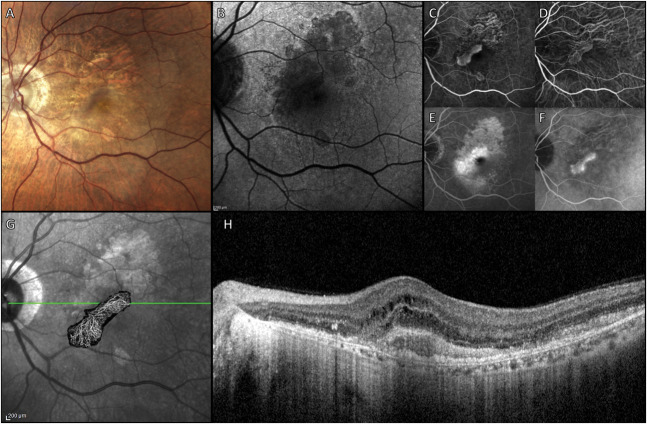
Multimodal imaging of Type 2 MNV in a patient with EMAP. **A.** A true-color fundus photograph shows large areas of RPE atrophy with a predominant vertical axis, surrounded by several pseudodrusen-like lesions. A loss of the foveal reflex is visible just inferior to the RPE atrophy. **B.** SW-AF reveals moderately hypoautofluorescent areas corresponding to RPE atrophy, with a pronounced hypoautofluorescent signal superonasal to the fovea. **C.** Early-phase fluorescein angiography displays a hyperfluorescent neovascular membrane, with pronounced leakage in the late phase (**E**). Indocyanine green angiography (ICGA) highlights the neovascular network (**D**), with characteristic late-phase staining (**F**). Swept-source optical coherence tomography angiography, superimposed on the near-infrared image, provides further visualization of the neovascular network (**G**). The optical coherence tomography scan through the fovea shows significant neuroretinal thickening because of intraretinal cysts, subretinal hyperreflective material, subretinal neovascularization (**H**).

**Fig. 2.**
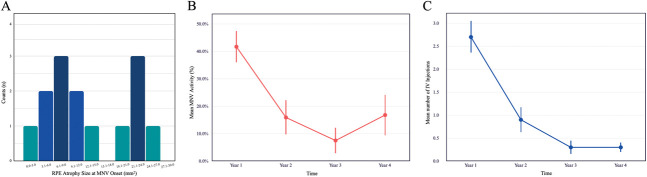
RPE atrophy size, lesion activity, and intravitreal injection frequency in eyes with EMAP complicated by MNV. **A.** A bimodal distribution of MNV development can be observed in relation to RPE atrophy size, with the first peak occurring between 6.1 mm^2^ and 9.0 mm^2^ and the second peak between 21.1 mm^2^ and 24.0 mm^2^. **B.** Mean lesion activity was highest in the first year (41.7%) and progressively declined over time, ranging from 7.5% to 16.8% in subsequent years. **C.** Similarly, the average number of intravitreal anti-VEGF injections decreased from 2.7 in the first year to 0.3 to 0.9 per year during years 2 to 4. Error bars in panels (**B** and **C**) represent the standard error of the mean.

Macular neovascularization was identified in 14 eyes (8.9%) of 10 patients, with bilateral involvement in 4 patients (40%). Macular neovascularization was present at baseline in eight eyes (57.1%). Macular neovascularization onset was associated with visual symptoms in 11 cases (78.6%). Most lesions were Type 2 (12 eyes, 85.7%) and primarily subfoveal (9 eyes, 64.3%) (Figure 1). Macular neovascularizations were active in 41.7% (SD: 23.9) of visits during the first year and responded well to anti-VEGF treatment, with a median of 2 (IQR: 1–3.8) injections needed in the first year and reactivations in four eyes (28.6%). Notably, three eyes (21.4%) experienced a significant BCVA decline attributable to fibrovascular scarring from MNV, while most cases had stable or improved vision after treatment. One eye (7.1%) developed an RPE tear after anti-VEGF treatment. Median RPE atrophy size at MNV onset was 9.1 mm^2^ (IQR: 6.2–22.1), with a bimodal distribution (peaks at 3.1–6.0 mm^2^ and 21.1–24.0 mm^2^; Figure 2). Specifically, MNV cases were classified as Stage 1 (five eyes, 35.7%), Stage 2 (six eyes, 42.9%), and Stage 3 (three eyes, 21.4%) (Table 2).

**Table 2.** Clinical and Imaging Features of EMAP Eyes Complicated by MNV

EMAP Eyes with MNV		
Eyes (patients)	14 (10)	
MNV types		
Type 1 n (%)	2 (14.3%)	
Type 2 n (%)	12 (85.7%)	
Type 3 n (%)	0 (0%)	
PCV n (%)	0 (0%)	
MNV location		
Subfoveal n (%)	9 (64.3%)	
Juxtafoveal n (%)	2 (14.3%)	
Extrafoveal n (%)	3 (21.4%)	
RPE atrophy size mm^2^, mean (SD) and median (IQR)	8.5 (9.3)	9.1 (6.2–22.1)
Anti-VEGF agent used		
Bevacizumab n (%)	7 (50.0%)	
Ranibizumab n (%)	5 (35.7%)	
Aflibercept n (%)	2 (14.3%)	
Time to fellow eye involvement months, mean (SD) and median (IQR)	13.6 (9.2)	11.5 (6.5–17.9)

PCV, polypoidal choroidal vasculopathy.

### Incidence and Factors Associated With Macular Neovascularization

**Fig. 3.**
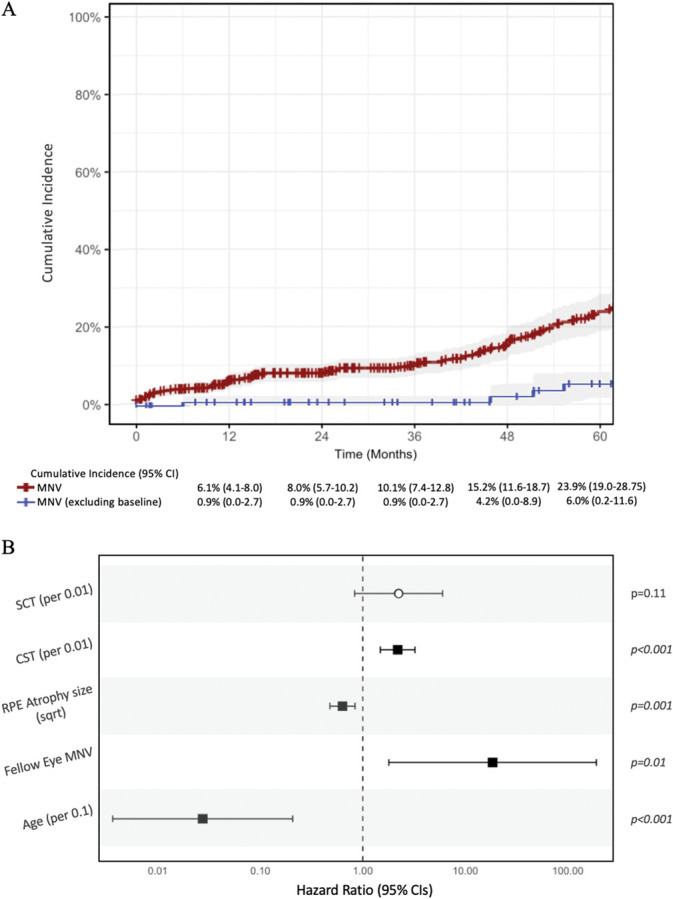
Cumulative incidence and risk factors for MNV development in EMAP. **A.** The Kaplan–Meier curve demonstrates a steady increase in MNV cases over time, with a cumulative incidence of 6.1% (95% CI: 4.1%–8.0%) at 1 year, rising to 15.2% (95% CI: 11.6%–18.7%) by year 4 (red curve). Excluding left-censored data, cumulative incidence was estimated at 0.9% (95% CI: 0.0–2.7) at 1 year and 4.2% (95% CI: 0.0–8.9) at 4 years (blue curve). The 95% confidence intervals are shaded in gray. **B.** Forest plots depict HR and 95% confidence intervals from a multivariable mixed-effects Cox regression analysis, identifying significant risk factors for MNV development. Notably, greater central subfield thickness (CST; *P* < 0.001), smaller RPE atrophy size (*P* = 0.001), fellow eye involvement (*P* = 0.01), and younger age (*P* < 0.001) were all significantly associated with an increased risk of MNV. CI, confidence interval.

Over a mean follow-up of 40.4 months, MNV developed in 14 eyes, yielding a cumulative incidence of 6.1% (4.1–8.0) at 1 year and 15.2% (11.6–18.7) at 4 years. Excluding left-censored data, the cumulative incidence was 0.9% (0.0–2.7) at 1 year and 4.2% (0.0–8.9) at 4 years. The median incidence rate was 2.3 (1.0–3.6) events per 100 person-years (1.2 [0.2–2.2] after excluding left-censored data). The Kaplan–Meier curve of MNV incidence is shown in Figure 3A.

Univariable Cox regression revealed that younger age (per 0.1; HR = 0.001, *P* < 0.001), fellow eye involvement (HR = 2.1, *P* < 0.001), smaller RPE atrophy size (HR = 0.51, *P* < 0.001), greater CST (HR = 2.1, *P* = 0.002), and greater subfoveal choroidal thickness (per 0.01; HR = 19.1, *P* < 0.001) were associated with a higher MNV risk. Multivariable Cox regression confirmed associations with younger age (HR = 0.03, *P* < 0.001), fellow eye involvement (HR = 18.4, *P* = 0.01), smaller RPE atrophy size (HR = 0.63, *P* = 0.001), and greater CST (per 0.01; HR = 2.2, *P* = 0.002) (Figure 3B and Table 3).

**Table 3.** Mixed-Effects Cox Proportional Hazards Regression Analysis Assessing the Relationship Between Demographic Features, Imaging Biomarkers, and Time-dependent Risk of Developing MNV in Eyes Affected by EMAP

Covariates	Cox PH Regression			
	Univariable Analysis		Multivariable Analysis	
	HR (95% CI)	*P*	HR (95% CI)	*P*
Age (0.1)	0.001 (0.0001–0.003)	*<0.001*	0.03 (0.004–0.21)	*<0.001*
Smoking status	2.40 (0.49–11.81)	0.28	—	—
Fellow eye involvement	2.14 (1.48–3.07)	*<0.001*	18.45 (1.79–189.75)	*0.01*
SW-AF atrophy borders	1.39 (0.08–25.51)	0.82	—	—
RPE atrophy size (square-rooted)	0.49 (0.45–0.54)	*<0.001*	0.63 (0.48–0.84)	*0.001*
VMID	2.39 (0.00–5.79)	0.07	—	—
ORT	0.49 (0.23–1.04)	0.07	—	—
CST (0.01)	2.06 (1.30–3.26)	*0.002*	2.19 (1.48–3.22)	*<0.001*
SCT (0.01)	19.06 (7.11–51.08)	*<0.001*	2.23 (0.83–6.00)	0.11

Significant *P*-values are in italics.

CI, confidence intervals; ORT, outer retinal tubulations; PH, proportional hazards; SCT, subfoveal choroidal thickness; SW-AF, short-wavelength autofluorescence; VMID, vitreomacular interface disorder.

### Impact of Macular Neovascularization on Best-Corrected Visual Acuity and Retinal Pigment Epithelium Atrophy

Eyes with MNV had lower baseline BCVA compared with those without MNV (58.4 vs. 71.4 ETDRS letters, approximately 20/63 vs. 20/40 Snellen; β= −12.9, *P* = 0.005). However, after adjusting for age and baseline BCVA, the rate of BCVA decline was similar between groups (−3.9 vs. −4.1 ETDRS letters/year; β = 0.24, *P* = 0.69).

**Fig. 4.**
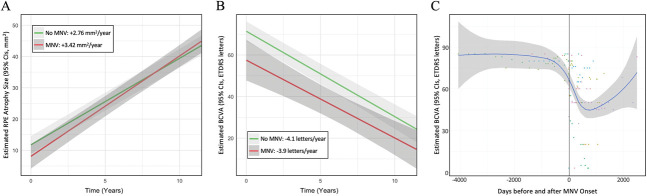
Estimated changes in RPE atrophy size and BCVA between eyes with and without MNV over time. **A.** After adjusting for age and baseline areas, eyes with MNV demonstrated a significantly higher rate of RPE atrophy progression compared with eyes without MNV (3.42 vs. 2.76 mm^2^/year, *P* = 0.02). **B.** After adjusting for age and baseline BCVA, no difference in the rate of BCVA decline was observed between eyes with and without MNV (−3.9 vs. −4.1 ETDRS letters/year, *P* = 0.69). **C.** This panel illustrates the predicted trend of BCVA changes around the onset of MNV, showing a marked decline in BCVA followed by visual improvement after anti-VEGF treatment.

**Fig. 5.**
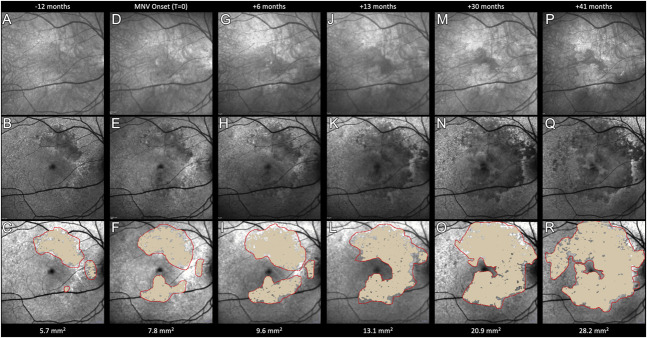
Longitudinal measurement of RPE atrophy in an eye with EMAP, complicated by MNV, from 12 months before its onset (**A**–**C**) to the final follow-up (**P**–**R**). The first row shows near-infrared images used to improve the delineation of RPE atrophy. The second row illustrates the progression of atrophy over time, as captured by SW-AF. The third row depicts the measured RPE atrophy area using the expert modus of the RegionFinder software (version 2.6.4; Heidelberg Engineering GmbH), with atrophic areas highlighted in yellow and constraints marked in red. SW-AF, short-wavelength autofluorescence.

At baseline, eyes with MNV had a moderately smaller, although not significant, RPE atrophy size (5.6 vs. 11.9 mm^2^; β = −0.25, *P* = 0.27). After adjusting for age and baseline RPE atrophy size, eyes with MNV showed a faster rate of RPE atrophy growth compared with those without MNV (3.4 vs. 2.8 mm^2^/year; β = 0.05, *P* = 0.02). Changes in BCVA and RPE atrophy size over time are shown in Figure 4. Figure 5 illustrates RPE atrophy measurements over time in an eye with MNV.

No significant associations were found between MNV lesion activity and the rates of RPE atrophy growth (β = 0.002, *P* = 0.88) or BCVA loss (β = 0.14, *P* = 0.12).

## Discussion

This study retrospectively analyzed the incidence, clinical features, and risk factors for MNV in a large Italian cohort of 79 EMAP patients. Most MNV cases were Type 2, predominantly subfoveal, and demonstrated a favorable anatomical response to anti-VEGF treatment. We also evaluated the impact of MNV on visual outcomes and RPE atrophy progression. While MNV had limited impact on BCVA after treatment, it was associated with a significantly faster rate of RPE atrophy growth in affected eyes.

Extensive macular atrophy with pseudodrusen-like appearance is an increasingly recognized retinal degeneration leading to legal blindness within 5 years from onset.^1,2,10,12^ Visual loss is generally because of foveal RPE atrophy or subfoveal non-neovascular fibrosis,^5^ although MNV can occasionally worsen visual outcomes. Previous studies on MNV in EMAP are limited and mostly consist of isolated case reports.^12–15^ Kamami-Levy et al^15^ reported choroidal neovascularization in approximately 10% of their cohort (four eyes from three patients), with good anatomical but limited functional response to anti-VEGF or laser treatment. More recently, Antropoli et al^12^ identified choroidal neovascularization in 19 cases (14.7%) in a large French cohort, although extra-macular cases were included and clinical outcomes were not assessed.

In our study, MNV developed in 14 eyes over a mean follow-up of ∼40 months, with a cumulative incidence of 6.1% at 1 year and 15.2% at 4 years for this complication (0.9% and 4.2% after excluding baseline MNV). These rates align with those reported by Kamamy-Levy and Antropoli,^12,15^ and with the prevalence of exudative MNV in geographic atrophy from AMD.^29,30^ Although MNV incidence in late-onset retinal degeneration—a phenocopy of EMAP—remains uncertain,^31^ the rates in our cohort are notably lower than in other phenocopies like pseudoxanthoma elasticum (42%–86%) and Sorsby fundus dystrophy (60%–80%).^32,33^ The high incidence in pseudoxanthoma elasticum may reflect BrM degradation and disrupted RPE-choriocapillaris communication, leading to VEGF upregulation,^34,35^ while differences with Sorsby fundus dystrophy suggest distinct angiogenic mechanisms in EMAP despite imaging similarities.

Most MNV cases in our study were Type 2 (85.7%) and subfoveal (64.7%) lesions, consistent with patterns seen in geographic atrophy and inherited EMAP phenocopies.^15,29^ This may reflect the significant EMAP-associated RPE alterations, which compromise the barrier function and promote subretinal invasion. Interestingly, unlike AMD,^36^ we found no Type 3 MNV cases, despite the presence of pseudodrusen-like deposits. Table 4 summarizes the clinical and MNV characteristics of EMAP and its phenocopies. Although treatment protocols varied, all MNV cases responded well to anti-VEGF injections on a pro re nata basis. The median number of injections in the first year was two, with fewer reactivations over time—suggesting pro re nata may be an effective approach for MNV in EMAP.

**Table 4.** Clinical and MNV Characteristics in EMAP and Key Phenocopies

	EMAP (Our Series)	GA	PXE	SFD	L-ORD
Large Drusen	Absent or rare	Present (sometimes regressed)	Absent	Present from third decade, also nasal to the ONH and along vascular arcades (Sivaprasad S et al. *Am J Ophthalmol* 2008) (Gliem M et al *IOVS* 2015)	Absent (Duncan HJ et al. *BMJ Open Ophthalmol* 2023)
RPD/RPD-like lesions	100%, with panretinal distribution (Hamel C et al. *Am J Ophthalmol*)	>60% (Schmitz-Valckenberg S et al. *IOVS* 2011)	〜50% (age-dependent) (Gliem M et al. *JAMA Ophthalmol* 2015)	>70% after sixth decade (Gliem M et al *Ophthalmology* 2015)	100%, from fifth decade (Borooah S et al. *Ophthalmol Retina* 2021)
BrM ruptures	25% of late-stage cases (Antropoli et al. *Ophthamology* 2024)	16%, especially in the presence of RPD (Sacconi R et al. *Ophthalmol Ther* 2023)	85%–95% (Finger RP et al. *Surv Ophthalmol* 2009)	Rare (Capon MR et al. *Ophthalmology* 1989)	Not documented
Choroidal thickness	Variable at baseline, rapidly thinning (including atrophy) (Romano F et al. *Ophthalmol Sci* 2021)	Thin (especially in diffuse-trickling SW-AF phenotype) (Lindner M et al. *IOVS* 2015)	Thinner in the presence of CNV or macular atrophy (Hidalgo-Dìaz T et al. *Int Ophthalmol* 2020)	Thinner with the onset of macular atrophy (Gliem M et al *IOVS* 2015)	Thin and rapidly thinning (Borooah S et al. *Retina* 2021)
MNV frequency	〜15% by 4 years	〜11% by 4 years (Sunness JS et al. *Ophthalmology* 1999) (Sacconi R et al. *Br J Ophthalmol* 2022)	42%–86% (Risseeuw S et al. *Retina* 2019)	60%–80% (Sivaprasad S et al. *Am J Ophthalmol* 2008)	No definitive studies
MNV types	Type 2 >> Type 1; no reports of Type 3, PCV, and nonexudative MNV	Type 2 >> Type 1 > Type 3. Absent/rare PCV. No definitive studies on nonexudative MNV frequency (Sacconi R et al. *Br J Ophthalmol* 2022)	Type 1 > Type 2; no reports of Type 3; isolated reports of PCV; nonexudative MNV (33%) (Cicinelli MV et al. *Ophthalmol Retina* 2024) (Marques JP et al. *Graefes Arch Clin Exp Ophthalmol* 2021)	Type 2 > Type 1 and PCV; no reports of Type 3 and nonexudative MNV (Sivaprasad S et al. *Am J Ophthalmol* 2008) (Gliem M et al. *IOVS* 2015)	No definitive studies: both Type 1 and 2 MNV sporadically described (Jeffery RCH et al. *Surv Ophthalmol* 2024)
MNV location	Subfoveal >> extrafoveal ≥ juxtafoveal	Subfoveal >> juxta-/extrafoveal (Sacconi R et al. *Br J Ophthalmol* 2022)	Subfoveal > extrafoveal > juxtafoveal (Rohart C et al. *Retina* 2023)	Sub-/juxtafoveal > extrafoveal (Sivaprasad S et al. *Am J Ophthalmol* 2008)	No definitive studies: typically, temporal to the fovea (Jeffery RCH et al. *Surv Ophthalmol* 2024)
MNV-atrophy relationship	Faster progression of atrophy	Slower local progression of atrophy (Pfau M et al. *Ophthalmol Retina* 2020)	Atrophy associated with Type 2 MNV onset and greater extent of AS (Risseeuw S et al. *Am J Ophthalmol* 2020) (Gliem M et al. *IOVS* 2016)	Unknown; SFD cases without MNV may have faster progression of atrophy	Unknown

AS, angioid streaks; BrM, Bruch membrane; GA, geographic atrophy; L-ORD, late-onset retinal degeneration; MNV, macular neovascularization; PCV, polypoidal choroidal vasculopathy; PXE, pseudoxanthoma elasticum; RPD, reticular pseudodrusen; SFD, Sorsby fundus dystrophy; SW-AF, short-wavelength autofluorescence.

Furthermore, Cox regression analysis identified several factors associated with MNV development in EMAP. The increased risk with fellow eye involvement may indicate strong intereye dependence, similar to AMD.^37^ However, the associations with younger age, smaller RPE atrophy size, and greater CST suggest that neovascular complications are more likely in the earlier stages of EMAP. This may represent a para-physiologic response to support hypoxic photoreceptors and locally inhibit RPE atrophy progression, as proposed in AMD.^38^ We also observed a bimodal distribution of MNV occurrence by RPE atrophy size, with peaks around 3.1 mm^2^ to 6.0 mm^2^ (Stage 1) and 21.1 mm^2^ to 24.0 mm^2^ (transition to Stage 3). The first peak may reflect increased VEGF production from viable RPE in response to the diffuse RPE-BrM separation acting as a barrier to photoreceptor nutrition.^7,9^ The second peak could indicate a late attempt to protect the fovea or result from BrM ruptures, which have been reported in 25% of late-stage EMAP cases.^12^ Based on these findings, and the asymptomatic nature of MNV in some cases, we recommend closer monitoring during Stages 1 and 2, particularly for patients with fellow eye involvement.

Finally, the recent Food and Drug Administration approval of anticomplement treatments for geographic atrophy has sparked interest in their potential application to other forms of macular atrophy.^39^ This is especially relevant given EMAP's resemblance to the diffuse-trickling phenotype of geographic atrophy and the altered complement levels detected in the initial French cohort.^4,6^ However, since anticomplement therapy in AMD has been associated with higher MNV rates,^40,41^ understanding the clinical impact of MNV in EMAP—beyond its incidence—becomes essential. While MNV was associated with worse BCVA at presentation (59 vs. 71 ETDRS letters, approximately 20/63 vs. 20/40 Snellen), we found no significant difference in BCVA decline over time between EMAP eyes with and without MNV. In contrast, RPE atrophy progression was faster in MNV eyes (3.4 vs. 2.8 mm^2^/year), even after adjusting for baseline size, diverging from previous clinical and histologic findings in AMD.^21,30,42^ Potential explanations for accelerated atrophy in MNV eyes include 1) MNV-related fibro-atrophic changes, particularly because of the high prevalence of Type 2 lesions; 2) possible side effects of physiologic VEGF levels suppression, although patients received few injections and lesion activity was not correlated with atrophy growth; and 3) a more aggressive EMAP phenotype characterized by MNV and rapid atrophy progression. Studying untreated EMAP cohorts may help clarify this aspect. Yet, we cannot rule out that current models using square-root transformation and adjustment for baseline size may not fully capture the exponential growth of macular atrophy in EMAP, suggesting a need for alternative regression models for atrophy assessment.

It is important to acknowledge some limitations, particularly the retrospective design, which prevented standardized follow-up and treatment protocols across centers. While the number of MNV cases was relatively small (14), MNV is a rare complication of EMAP, and our cohort remains one of the largest with longitudinal follow-up in the literature. In addition, functional tests such as microperimetry may correlate more closely with EMAP stages than BCVA alone.^11^ Future studies incorporating functional assessments beyond BCVA may offer a more nuanced understanding of MNV's impact on visual function. We also did not assess extra-macular neovascularization because of the lack of standardized widefield imaging, and nonexudative Type 1 MNV may have been missed in the absence of routine longitudinal optical coherence tomography angiography assessments. Moreover, while BrM ruptures have recently emerged as a relevant biomarker in advanced EMAP, its low prevalence in our relatively younger cohort precluded its inclusion in the statistical analysis. We look forward to exploring the relationship between BrM ruptures and MNV in future prospective studies. Finally, measuring RPE atrophy using short-wavelength autofluorescence can be challenging in eyes with MNV.^22,43^ To minimize this, we used a validated method incorporating near-infrared imaging and structural OCT,^22^ which resulted in excellent intergrader agreement.

To conclude, we reported a 4-year cumulative incidence of 15.2% for MNV in a large Italian cohort of EMAP patients, identifying younger age and smaller RPE atrophy size as significant risk factors. The high prevalence of Type 2 lesions and the bimodal distribution of RPE atrophy size at MNV onset suggest two possible pathways in MNV pathogenesis: 1) early-stage involvement of a dysfunctional yet viable RPE layer and 2) BrM ruptures promoting neovascularization in more advanced stages. Clinically, while anti-VEGF treatment led to prompt BCVA improvement, RPE atrophy expanded more rapidly in MNV eyes, potentially because of fibrosis-related atrophic changes or a more aggressive disease phenotype. These findings provide critical insights into EMAP's natural history and may guide future targeted therapies aimed at slowing macular atrophy growth.

## Supplementary Material

SUPPLEMENTARY MATERIAL
