# Supplementary material for: INCIDENCE, CHARACTERISTICS, AND OUTCOMES OF MACULAR NEOVASCULARIZATION IN EXTENSIVE MACULAR ATROPHY WITH PSEUDODRUSEN-LIKE APPEARANCE
Source: Retina. 2025 Apr 30;45(9):1689–700. doi: 10.1097/IAE.0000000000004506 (PMC12366734; doi:10.1097/IAE.0000000000004506)
Supplement: SUPPLEMENTARY MATERIAL [file retina-45-1689-s001.pdf]

**Supplementary Digital Content 1. Inter-grader agreement for the graded qualitative and quantitative variables.**

| Qualitative Grading | |
|---|---|
| **Variable** | **k (95% CI)** |
| VMID Presence | 0.82 (0.78-0.85) |
| ORT Presence | 0.87 (0.84-0.90) |
| SW-AF Atrophy Borders | 0.78 (0.74-0.82) |
| MNV Type | 1.00 |
| MNV Location | 0.88 (0.86-0.91) |
| MNV Activity | 0.79 (0.75-0.83) |
| **Quantitative Grading** | |
| **Variable** | **ICC (95% CI)** |
| RPE Atrophy Size | 0.86 (0.82-0.90) |
| CST | 0.94 (0.91-0.96) |
| SCT | 0.84 (0.81-0.87) |

*Legend: k, Cohen's k-factor; CI, confidence intervals; VMID, vitreomacular interface disorder; ORT, outer retinal tubulations; SW-AF, short-wavelength autofluorescence; MNV, macular neovascularization; ICC, intra-class correlation coefficient; RPE, retinal pigment epithelium; CST, central subfield thickness; SCT, subfoveal choroidal thickness.*
